# Epstein-Barr virus BALF3 mediates genomic instability and progressive malignancy in nasopharyngeal carcinoma

**DOI:** 10.18632/oncotarget.2323

**Published:** 2014-08-10

**Authors:** Shih-Hsin Chiu, Chung-Chun Wu, Chih-Yeu Fang, Shu-Ling Yu, Hui-Yu Hsu, Yen-Hung Chow, Jen-Yang Chen

**Affiliations:** ^1^ Graduate Institute of Microbiology, College of Medicine, National Taiwan University, Taipei, Taiwan; ^2^ National Institute of Cancer Research, National Health Research Institutes, Miaoli, Taiwan; ^3^ National Institute of Infectious Diseases and Vaccinology, National Health Research Institutes, Miaoli, Taiwan

**Keywords:** nasopharyngeal carcinoma, relapse, Epstein-Barr virus, BALF3, genomic instability

## Abstract

Nasopharyngeal carcinoma (NPC) is a head and neck cancer prevalent throughout Southern China and Southeast Asia. Patient death following relapse after primary treatment remains all too common but the cause of NPC relapse is unclear. Clinical and epidemiological studies have revealed the high correlation among NPC development, Epstein-Barr virus (EBV) reactivation and host genomic instability. Previously, recurrent EBV reactivation was shown to cause massive genetic alterations and enhancement of tumor progression in NPC cells and these may be required for NPC relapse. Here, EBV BALF3 has the ability to induce micronuclei and DNA strand breaks. After recurrent expression of BALF3 in NPC cells, genomic copy number aberrations, determined by array-based comparative genomic hybridization, had accumulated to a significant extent and tumorigenic features, such as cell migration, cell invasion and spheroid formation, increased with the rounds of induction. In parallel experiments, cells after highly recurrent induction developed into larger tumor nodules than control cells when inoculated into NOD/SCID mice. Furthermore, RNA microarrays showed that differential expression of multiple cancer capability-related genes and oncogenes increased with recurrent BALF3 expression and these changes correlated with genetic aberrations. Therefore, EBV BALF3 is a potential factor that mediates the impact of EBV on NPC relapse.

## INTRODUCTION

Nasopharyngeal carcinoma (NPC) is an endemic cancer, which is distributed predominantly through certain geographic areas and in particular racial/ethnic populations. In Southern China and Southeast Asia, especially among Cantonese men, the incidence rate of NPC is approximately 10 to 50 per 100,000 person-years; in addition, it also occurs sporadically in other parts of the world, including native populations in Canada, Greenland, Alaska, the Middle East and North Africa [1, 2]. The endemic nature, as well as carcinogenesis, of this disease are considered a consequence of Epstein-Barr virus (EBV) infection, together with environmental and host genetic factors. To date, the 5-year survival rate of locally advanced NPC patients can reach approximately 70% after primary treatment. However, the incidence rates of local-regional relapse and distant metastasis, both of which are the major risks of patient death, are still more than 20% [3-7] and the cause remains enigmatic.

EBV is a human γ-herpesvirus that infects more than 90% of the adult population worldwide. EBV is a causative agent of infectious mononucleosis and is highly associated with several human malignancies, including NPC. The etiological association of NPC with EBV infection has been strongly supported by various clinical and epidemiological studies. Viral DNA and lytic gene products such as early antigen diffuse (EA-D), early antigen restricted (EA-R), BZLF1, BRLF1, BMLF1, gp220, lytic latent membrane protein 1, DNase, membrane antigen, viral capsid antigen (VCA) and BALF1 may be detected in NPC biopsies [8-14]. Serological studies have reported that patients with NPC generally exhibit high levels of IgA antibodies against EBV EA and VCA [15-21] and elevated titers of neutralizing antibodies against EBV DNA replication-related enzymes such as DNase, DNA polymerase and thymidine kinase also are detectable in the sera of patients with this disease [21-28]. Moreover, the titers of these antibodies increase with the stage of NPC [16-18, 28] and patients with remission after effective therapy show a decline of the antibodies over time. However, a reversal to increasing titers is seen in long-term survivors with relapse or metastasis [16-18, 20, 23]. Therefore, these prospective and retrospective investigations suggest that the antibodies against EBV lytic antigens are indicative serological markers for the prognosis and diagnosis of NPC. Taking these observations together, EBV plays a critical role in the development of NPC.

Genomic instability is defined as an increase in the frequency of genetic alterations, ranging from the nucleotide to the chromosome level and including subtle sequence changes, chromosome number alterations, chromosome translocations and gene amplifications. This is an early event and leads to the emergence of the characteristics of cancer in the cells [29, 30]. In NPC biopsies, conventional cytogenetic analyses show tumor cells with the appearance of polyploidy, aneuploidy and marker chromosomes [31, 32]. High frequencies of loss of heterozygosity (LOH) are observed for chromosomes 1p, 2p, 2q, 3p, 3q, 5q, 9p, 9q, 11q, 13q, 14q and 17q [33-39]; moreover, a genome-wide analysis utilizing comparative genomic hybridization (CGH) identifies that gains of genomic copy number aberrations (CNAs) cluster on chromosomes 1p, 1q, 2q, 3q, 5q, 6p, 6q, 7p, 7q, 8p, 8q, 9q, 11q, 12p, 12q, 15q, 17q, 18q, 19p, 19q, 20p and 20q and losses are among 1p, 3p, 5q, 9p, 9q, 11q, 13q, 14q and 16q [40-44]. In addition, the extent of these alterations is correlated with NPC stage, relapse and metastasis [39, 40, 43]. Accordingly, genomic instability occurs comprehensively in NPC and contributes to tumor progression.

Previously, we found that the EBV latent gene product LMP1 and EBNA2 are capable of inducing genomic instability in epithelial cells [45-48]. However, in studies of the clinical features of NPC, NPC patients with high titers of antibodies against EBV EA and VCA have tumor cells with a high frequency of LOH [39, 49]. Elevation of antibodies against EBV lytic gene products is considered to be a sign of reactivation of EBV [50-53]. Therefore, we investigated the lytic induction of EBV in NPC cell lines and high frequencies of significant increases of micronuclei and DNA double-strand breaks, relative to latency, were revealed. With recurrent reactivation of EBV, the occurrence of micronuclei and structural chromosomal aberrations accumulate in the cells and frequent CNAs are produced predominantly on chromosomes 3, 7, 8, 9 and X [54, 55]. These results suggest that EBV reactivation could cause genomic instability in the host cells and is an underlying factor of NPC relapse. Furthermore, EBV DNase and BGLF4, lytic gene products, were shown to be able to elicit genomic instability [56, 57]. EBV BALF3 is expressed after EBV reactivation and is a terminase, which cleaves newly synthesized viral DNA and enables the translocation of unit length genomes into procapsids during the lytic cycle [58]. In addition, our preliminary screening suggests that BALF3 is involved in the induction of host genomic instability. In this study, we examined EBV BALF3 expressed by recurrent induction to explore its effects not only on host genomes but also its tumorigenic properties in NPC cells and a genome-wide analysis was used to determine the differentially expressed genes involved, including cancer capability-related genes and oncogenes.

## RESULTS

### Effect of EBV BALF3 on genomic instability

**Figure 1:**
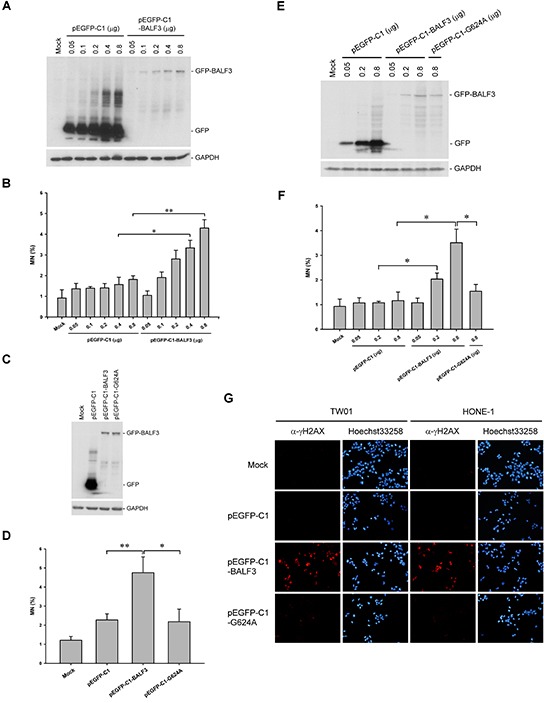
Induction of genomic instability in NPC cells with EBV BALF3 expression TW01 cells were transiently transfected with the doses of pEGFP-C1 or pEGFP-C1-BALF3 indicated for 24 h, followed by western blotting with antibodies specific to GFP and GAPDH **(A)** and micronucleus assay **(B)**. Data are presented as means ± standard deviations. Student's *t* test was used to determine the difference between two groups. *, *P* < 0.05; **, *P* < 0.001. Mock, mock transfection; MN, micronucleus. **(C** and **D)** TW01 cells were transiently transfected with 0.8 μg pEGFP-C1, pEGFP-C1-BALF3 or pEGFP-C1-G624A for 24 h prior to western blotting and micronucleus assay. Data are presented as means ± standard deviations. Student's *t* test was used to determine the difference between two groups. *, *P* < 0.05; **, *P* < 0.01. Mock, mock transfection; MN, micronucleus. **(E** and **F)** HONE-1 cells were transiently transfected with the doses of pEGFP-C1, pEGFP-C1-BALF3 or pEGFP-C1-G624A indicated for 24 h, following by western blotting and micronucleus assay. Data are presented as means ± standard deviations. Student's *t* test was used to determine the difference between two groups. *, *P* < 0.05. Mock, mock transfection; MN, micronucleus. **(G)** TW01 and HONE-1 cells were transiently transfected with 0.8 μg pEGFP-C1, pEGFP-C1-BALF3 or pEGFP-C1-G624A for 24 h prior to indirect immunofluorescence staining with an antibody specific to γH2AX. The nuclei of the cells were stained with Hoechst33258. Mock, mock transfection.

To determine whether EBV BALF3 (NCBI GenBank accession no. AFY97901) can cause genomic instability in host cells, TW01 cells were transiently transfected with pEGFP-C1-BALF3 and were examined for the formation of micronuclei, which arise during mitosis from acentric chromosome fragments and lagging whole chromosomes and are used as a biomarker for chromosome breakage and loss [59, 60]. A representative immunoblot shows that increasing GFP-BALF3 expression was detected in a dose-dependent manner (Figure 1A) and the numbers of micronuclei correlated with the expression kinetics of BALF3, increasing by up to approximately 1.0, 1.9, 2.8, 3.3 and 4.3% with pEGFP-C1-BALF3 at doses of 0.05, 0.1, 0.2, 0.4 and 0.8 μg, respectively (Figure 1B). The phosphorylation of serine 139 in the C-terminal tail of the histone H2A variant, γH2AX, is initiated in chromatin by DNA double-strand breaks (DSBs) [61]. The cells expressing GFP-BALF3 exhibited more H2AX phosphorylation than the mock and pEGFP-C1 transfections, detected by immunofluorescence staining with an antibody specific to γH2AX (Figure 1G), suggesting that EBV BALF3 could induce DSBs. Moreover, a recent study in our laboratory has shown that alanine substitution of the glycine residue at amino acid 624 of EBV BALF3 results in reduced endonucleolytic activity [58]. Therefore, the coding region of BALF3 in pEGFP-C1-BALF3 was mutated by replacement of glycine 624 with alanine, with the result that the occurrence of micronuclei and H2AX phosphorylation induced by the *G624A* mutant was reduced to a significant extent, compared to the wild-type transfection (Figure 1C, D and G), confirming that EBV BALF3 could generate chromosome breakage and loss and DSBs. These results were also seen in an additional NPC cell line, HONE-1 (Figure 1E, F and G). According to these observations, EBV BALF3 is able to induce genomic instability in host cells.

**Figure 2:**
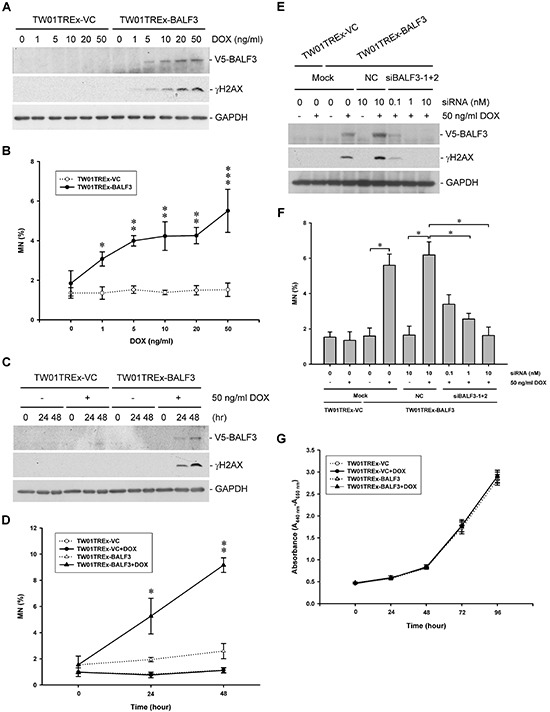
Effect of EBV BALF3 expression on genomic instability and growth of NPC cells TW01TREx-VC and TW01TREx-BALF3 cells were treated with the concentrations of DOX indicated for 24 h and subjected to western blotting with antibodies specific to V5, γH2AX and GAPDH **(A)** and micronucleus assay **(B)**. Data are presented as means ± standard deviations. Student's *t* test was used to determine the difference between two groups. *, *P* < 0.05; **, *P* < 0.01; ***, *P* < 0.001, compared to TW01TREx-BALF3 cells without DOX treatment. DOX, doxycycline; MN, micronucleus. **(C** and **D)** TW01TREx-VC and TW01TREx-BALF3 cells were treated with 50 ng/ml DOX for 0, 24 and 48 h prior to western blotting and micronucleus assay. Data are presented as means ± standard deviations. Student's *t* test was used to determine the difference between two groups. *, *P* < 0.05; **, *P* < 0.001, compared to TW01TREx-BALF3 cells at 0 h post-induction. DOX, doxycycline; MN, micronucleus. **(E** and **F)** TW01TREx-VC and TW01TREx-BALF3 cells were transiently transfected with 10 nM Negative Control Duplexes (NC) or the concentrations of siBALF3-1 and -2 indicated prior to 50 ng/ml DOX treatment for 24 h, followed by western blotting and micronucleus assay. Data are presented as means ± standard deviations. Student's *t* test was used to determine the difference between two groups. *, *P* < 0.05. Mock, mock transfection; DOX, doxycycline; MN, micronucleus. **(G)** TW01TREx-VC and TW01TREx-BALF3 cells were treated with 50 ng/ml DOX for 0, 24, 48, 72 and 96 h and the numbers of viable cells were determined by WST-1 reagent. Data are presented as means ± standard deviations. DOX, doxycycline.

For long-term expression experiments, we established an EBV BALF3-inducible NPC cell line, TW01TREx-BALF3, which harbors the BALF3 coding region with a V5 tag, the expression of this gene product being induced by DOX treatment. Comparing the physical properties of TW01TREx-VC and -BALF3 cells, TW01TREx-BALF3 cells showed more micronuclei and H2AX phosphorylation after induction and both of the DNA damage indicators were increased with DOX at concentrations from 0 to 50 ng/ml (Figure 2A and B). After more than two rounds of the host cell cycle, the accumulation of DNA damage was clear and the cell population with micronuclei increased to approximately 9.2% at 48 h post-induction in TW01TREx-BALF3 cells (Figure 2C and D). The combination of siBALF3-1 and -2 for gene silencing verified the specific effect of EBV BALF3 on DNA damage (Figure 2E and F). In addition, under DOX induction at the maximal concentration of 50 ng/ml, there was no obvious cytotoxicity in the cells up to 96 h post-induction (Figure 2G). Therefore, this inducible cell line provides further evidence supporting the effect of EBV BALF3 on genomic instability and is a tool for further long-term studies.

### Accumulation of genomic instability after recurrent expression of EBV BALF3

**Figure 3:**
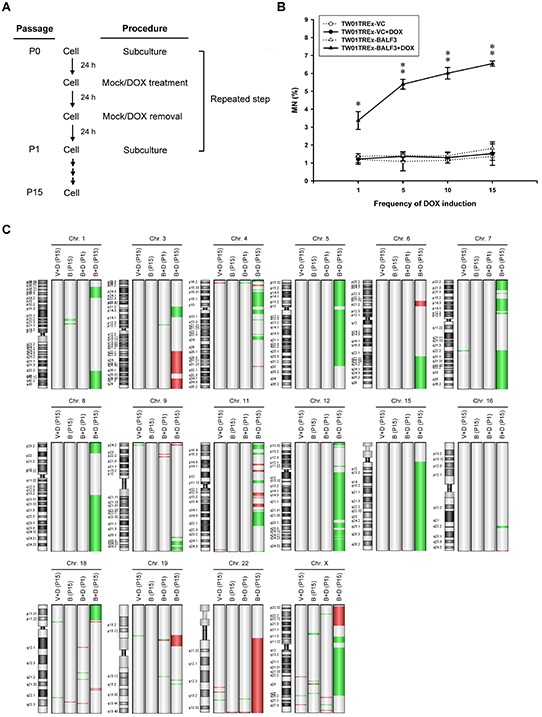
Accumulation of genomic instability in NPC cells after recurrent EBV BALF3 expression **(A)** Representative illustration of recurrent EBV BALF3 expression in NPC cells. The recurrent EBV BALF3 expression protocol was as follows. Cells were seeded on a culture plate and incubated for 24 h, followed by mock treatment or treatment with 5 ng/ml DOX for 24 h. After incubation, the culture medium was replaced and incubated for 24 h prior to cell subculture for the next round. These above procedures were defined as one passage (P) and repeated. The cycle was carried out up to 15 rounds. Mock, mock treatment; DOX, doxycycline; P, passage. **(B)** The cells were harvested at passages 1, 5, 10 and 15 and subjected to micronucleus assay. Data are presented as means ± standard deviations. Student's *t* test was used to determine the difference between two groups. *, *P* < 0.05; **, *P* < 0.01, compared to TW01TREx-BALF3 cells without DOX treatment. DOX, doxycycline; MN, micronucleus. **(C)** The genomic DNA of cells at passages 1 and 15 were extracted and subjected to array CGH and TW01TREx-BALF3 (P1) was used as a common reference. Images were produced by Agilent Genomic Workbench version 7.0.4.0. The location of amplifications and deletions of each group was displayed to the right side of the graph of a chromosome with cytobands. Red and green colors indicate amplification and deletion, respectively. V+D, TW01TREx-VC+DOX; B, TW01TREx-BALF3; B+D, TW01TREx-BALF3+DOX; DOX, doxycycline; P, passage; Chr, chromosome.

Because EBV reactivation may occur periodically prior to NPC relapse [62], the expression of EBV BALF3 was induced and recovered to a basal level repeatedly in TW01TREx-BALF3 cells, with DOX treatment and removal, to imitate the natural situation. The experimental protocol is illustrated in Figure 3A. DOX induction at a concentration of 5 ng/ml for 24 h led to BALF3 expression and the decrease followed removal of the inducer for a further 24 h, determining the procedure for recurrent EBV BALF3 expression in this study (Supplementary Figure S1). Following this protocol, recurrent induction was carried out over 15 passages and cells at passages 1, 5, 10 and 15 were harvested for further studies. The detection of micronuclei in TW01TREx-BALF3 cells after induction increased by up to approximately 3.7, 5.4, 6.0 and 6.5% for passages 1, 5, 10 and 15, respectively (Figure 3B). Similarly, micronucleus formation increased more in HONE-1 cells by five rounds of transfection of pEGFP-C1-BALF3 than the single transfection and the controls (Supplementary Figure S2). Furthermore, array CGH analysis was used for surveillance of CNAs on the host genome to investigate the effect of recurrent EBV BALF3 expression on genetic alterations. Here, this analysis was applied to TW01TREx-VC+DOX (P15) (TW01TREx-VC cells with DOX treatment harvested at passage 15), TW01TREx-BALF3 (P1), TW01TREx-BALF3 (P15), TW01TREx-BALF3+DOX (P1) and TW01TREx-BALF3+DOX (P15). Compared to TW01TREx-BALF3 (P1), a common reference, a clear increase of CNAs occurred in TW01TREx-BALF3+DOX (P15) and these were seen on chromosomes 1, 3, 4, 5, 6, 7, 8, 9, 11, 12, 15, 16, 18, 19, 22 and X; in contrast, relatively few CNAs were detected in TW01TREx-VC+DOX (P15), TW01TREx-BALF3 (P15) and TW01TREx-BALF3+DOX (P1) (Figure 3C). Data filtering of array CGH with high aberration scores, > 10 for amplification and < -10 for deletion, reveals high-level gains of altered loci in the TW01TREx-BALF3+DOX (P15) genome, located on chromosomes 3, 6, 11, 19, 22 and X, and losses were among chromosomes 1, 3, 4, 5, 6, 7, 8, 9, 11, 12, 15, 16, 18 and X, whereas TW01TREx-VC+DOX (P15), TW01TREx-BALF3 (P15) and TW01TREx-BALF3+DOX (P1) had no significant aberrations. These data are summarized in Table 1. In addition, there was no apparent CNA generation under DOX treatment, single induction of BALF3 expression and long-term cell culture. From the results of micronucleus assay coupled with array CGH analysis, EBV BALF3 with recurrent expression may be seen to play an important role in inducing aggravated genomic instability in the cells.

**Table 1:** Summary of array CGH data with high aberration score^*a*^

Chr. (no. of altered loci)		
Cell group	Amplification	Deletion
TW01TREx-VC+DOX (P15)	ND	ND
TW01TREx-BALF3 (P15)	ND	ND
TW01TREx-BALF3+DOX (P1)	ND	ND
TW01TREx-BALF3+DOX (P15)	3 (246), 6 (77), 11 (98), 19 (85), 22 (419), X (103)	1 (512), 3 (125), 4 (173), 5 (537), 6 (221), 7 (467), 8 (353), 9 (80), 11 (172), 12 (638), 15 (550), 16 (15), 18 (43), X (363)

Abbreviations: array CGH, array-based comparative genomic hybridization; Chr, chromosome; DOX, doxycycline; ND, not detected.

a The group of TW01TREx-BALF3 (P1) was used as a common reference. The data of array CGH were filtered by high aberration scores as follows: > 10 for amplification; < −10 for deletion.

### Contribution of EBV BALF3 to the tumorigenic properties of NPC cells

**Figure 4:**
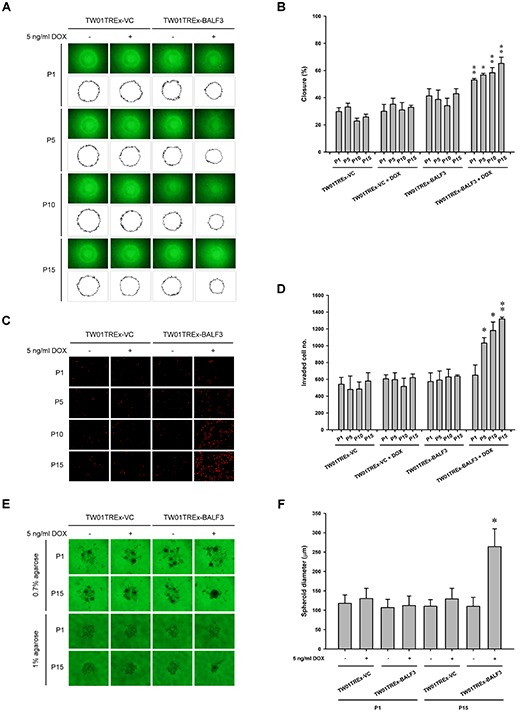
Examination of cell migration and invasion and spheroid formation by NPC cells after recurrent EBV BALF3 expression Treatment of TW01TREx-VC and TW01TREx-BALF3 cells was performed according to recurrent EBV BALF3 expression protocol. **(A)** The cells were harvested at passages 1, 5, 10 and 15 and subjected to cell migration assay for 12 h incubation. The area of a cell-free zone was measured by ImageJ. **(B)** Cell migration was determined as percent closure and calculated as described in Materials and Methods. Data are presented as means ± standard deviations. Student's *t* test was used to determine the difference between two groups. *, *P* < 0.01; **, *P* < 0.001, compared to TW01TREx-BALF3 cells without DOX treatment. DOX, doxycycline; P, passage. **(C)** The cells were harvested at passages 1, 5, 10 and 15 and subjected to cell invasion assay for 12 h incubation and the invaded cells were visualized using propidium iodide staining. **(D)** The numbers of invaded cells were counted and the data are presented as means ± standard deviations. Student's *t* test was used to determine the difference between two groups. *, *P* < 0.01; **, *P* < 0.001, compared to TW01TREx-BALF3 cells without DOX treatment. DOX, doxycycline; P, passage. **(E)** The cells were harvested at passages 1 and 15 and subjected to spheroid assay with 0.7 or 1% agarose matrix for 21 days incubation. **(F)** The diameter of spheroids was measured from images of 0.7% agarose group. Data are presented as means ± standard deviations. Student's *t* test was used to determine the difference between two groups. *, *P* < 0.001, compared to TW01TREx-BALF3 cells without DOX treatment at passage 15. DOX, doxycycline; P, passage.

Most cancers acquire a number of specific attributes during their development, including tissue invasion and metastasis [63]. To determine whether recurrent EBV BALF3 expression may lead to the malignant development of NPC cells, the repeatedly induced cell lines were analyzed for their tumorigenic phenotypes, including cell migration, cell invasion and spheroid formation. TW01TREx-BALF3 cells with DOX induction were subjected to cell migration and invasion assays and the percentage of closure increased by up to approximately 53.0, 56.7, 58.4 and 65.2% for passages 1, 5, 10 and 15, respectively, relative to the baseline of each group (Figure 4A and B). In addition, the average number of invading cells at passages 1, 5, 10 and 15 increased by up to approximately 649, 1032, 1180 and 1319, respectively (Figure 4C and D). Therefore, TW01TREx-BALF3 cells with DOX induction exhibit more migratory and invasive behavior than the TW01TREx-VC cell groups and cells with mock induction and there is a high correlation between these aggravated properties and the number of rounds of induction.

Most cells are present in three-dimensional structures in the human body and the formation of multicellular spheroids represents a tissue-like architecture and models the response to cell migration and invasion, as well as intercellular adhesion. Here, in a matrix of 0.7% agarose, TW01TREx-BALF3+DOX (P15) cells formed spheroids with a diameter of 264.2 μm, which was larger than those of TW01TREx-BALF3+DOX (P1), TW01TREx-VC+DOX (P1), TW01TREx-VC+DOX (P15) and the groups with mock induction (Figure 4E and F). Furthermore, the TW01TREx-BALF3+DOX (P15) cells were examined in a harder matrix, 1% agarose, and aggregated into a cluster, whereas the cells of the other groups were dispersed on the surface of the matrix (Figure 4E). These results suggest that the potential for intercellular adhesion, in addition to cell migration and invasion, was enhanced by recurrent EBV BALF3 expression.

**Figure 5:**
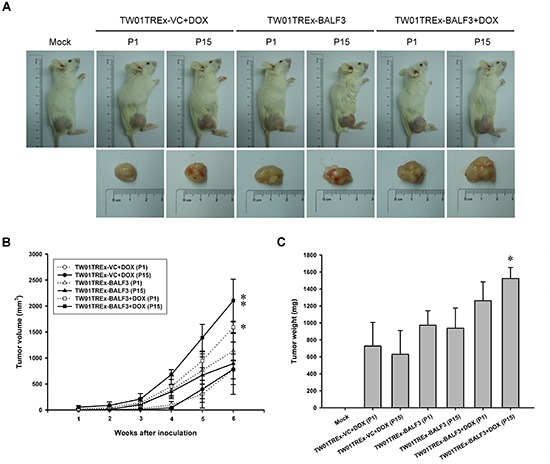
Examination of *in vivo* tumor growth after recurrent EBV BALF3 expression Treatment of TW01TREx-VC and TW01TREx-BALF3 cells was performed according to the recurrent EBV BALF3 expression protocol. The cells at passages 1 and 15 were injected subcutaneously into NOD/SCID mice and four animals per group were studied and observed for 6 weeks. **(A)** Sacrificed mice and tumor nodules after excision were photographed at week 6. Mock, mock inoculation; DOX, doxycycline; P, passage. **(B)** The tumor size was measured weekly by callipers and calculated as described in Materials and Methods. Data are presented as means ± standard deviations. Student's *t* test was used to determine the difference between two groups. *, *P* < 0.05; **, *P* < 0.01, compared to TW01TREx-BALF3 cells without DOX treatment. DOX, doxycycline; P, passage. **(C)** The tumor nodules were weighed after excision from mice sacrificed at week 6. Data are presented as means ± standard deviations. Student's *t* test was used to determine the difference between two groups. *, *P* < 0.01, compared to TW01TREx-BALF3 cells without DOX treatment at passage 15. Mock, mock inoculation; DOX, doxycycline; P, passage.

Based on the dramatically increased tumorigenic properties of NPC cells induced by recurrent EBV BALF3 expression observed above, the TW01TREx-VC+DOX, TW01TREx-BALF3 and TW01TREx-BALF3+DOX cells at passages 1 and 15 were injected subcutaneously into NOD/SCID mice to evaluate the effect of recurrent EBV BALF3 expression on tumor growth. Weekly monitoring of tumor growth revealed that tumors were formed by all of the cell groups and the size and weight of tumor nodules from TW01TREx-BALF3+DOX (P15) cells were up to approximately 2105.1 mm^3^ and 1523.3 mg, respectively, at week 6 post-inoculation and developed more actively than the other cell groups (Figure 5A to C). Therefore, highly recurrent induction of EBV BALF3 may contribute to the progressive malignancy of tumors.

### Differential expression of cancer-related genes in NPC cells after recurrent EBV BALF3 expression

**Figure 6:**
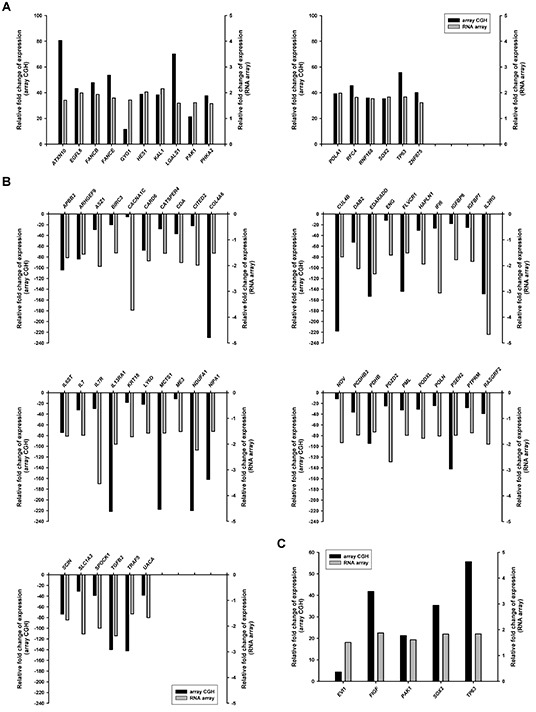
Differentially expressed genes in NPC cells after recurrent EBV BALF3 expression Treatment of TW01TREx-BALF3 cells was performed according to the recurrent EBV BALF3 expression protocol. The cells were subjected to array CGH and RNA expression array and TW01TREx-BALF3+DOX (P15) was compared to TW01TREx-BALF3 (P1). Genes shown in this figure were present in and cross-validated by data of array CGH and RNA array. The overexpressed **(A)** and underexpressed **(B)** cancer capability-related genes in Table S1 and the oncogenes **(C)** were common in both sets of array results.

To explore the changes in gene expression that result from repeated induction of EBV BALF3 expression, the various cell lines with repeated induction were analyzed using an RNA expression array. Using the criteria of absolute fold changes > 1.5 and *P* value < 0.05, the profile of TW01TREx-BALF3+DOX (P15) features 991 differentially expressed genes, including 428 overexpressed and 563 underexpressed genes, compared to TW01TREx-BALF3 (P1). The biological representation of these genes was unified by DAVID, a bioinformatics resource for functional annotation, and the terms of gene ontology set were related to capabilities for the characteristics of cancer, including cell adhesion, cell death, the immune system, cell migration, cell growth, vasculature development, precursor metabolites and energy generation, defense response, cell cycle, DNA repair and DNA damage response [63]. These sets included 172 altered genes (Supplementary Table S1). Furthermore, comparison of the RNA expression array and array CGH results revealed 62 genes which were differentially expressed in both. Among 62 altered genes, 16 were overexpressed and 46 were underexpressed and these are listed in Table S1 (Figure 6A and B). In addition, 5 oncogenes identified by NCBI (http://www.ncbi.nlm.nih.gov/gene) and in the relevant literature, *EVI1*, *FIGF*, *PAK1*, *SOX2* and *TP63* were overexpressed and present in the data sets of both arrays (Figure 6C). Therefore, recurrent expression of EBV BALF3 in NPC cells leads to differential expression of various cancer capability-related genes and oncogenes, which may be attributable to genetic aberrations.

## DISCUSSION

The detection of antibodies against EBV antigens in the sera of NPC patients was reported as early as 1966 [64]. So far, numerous clinical tests have shown that patients with NPC have EBV lytic gene products and IgA antibodies against EBV lytic antigens in tumor biopsies and sera, respectively; in addition, NPC tissues contain the linear form of viral DNA, which is consistent with the detection of replicative RNAs [65, 66]. Moreover, the titers of IgA antibodies against EBV EA, VCA and DNase are lower in the sera of patients with other EBV-associated diseases, such as infectious mononucleosis, Burkitt's lymphoma and Hodgkin's lymphoma, than with NPC [16, 22, 67]. Accordingly, these findings indicate significant levels of EBV reactivation in NPC. Furthermore, NPC also shows various limited expression patterns of EBV lytic genes, which account for an incomplete process of virion production [11, 65], and no viral particles can be detected in tumor tissues, despite the expression of lytic gene products and the increased titers of anti-lytic antigen IgA antibodies [9, 10, 12, 68]. This suggests that an abortive lytic cycle may occur in NPC and may protect the cells from death, which could provide an environment for the occult influence of EBV reactivation on the cells.

EBV BALF3 is required for viral DNA cleavage and packaging during the lytic cycle. To investigate the effect of BALF3 on uninfected cells, BALF3 was expressed alone in NPC cells and these were examined for genetic alterations using several methods, showing that the BALF3 expression led to the induction of micronuclei, DNA strand breaks and genomic CNAs (Figure 1, 2 and 3). BALF3 has nuclease activity and the BALF3-mediated nucleolytic reaction occurs not only in the terminal repeats of the EBV genome, which contain cleavage/packaging signals, but also *in vitro* in plasmid DNA without the terminal repeat sequence [58]. Similarly, nonspecific cleavage also has been reported for the terminases of human cytomegalovirus (HCMV) and bacteriophage T4. HCMV UL56 can cleave plasmid DNA *in vitro* in the absence of the *a* sequence and bacteriophage T4 gp17 carries out random cleavage of the *Escherichia coli* genomic DNA *in vivo* [69, 70]. In addition, Figure 1D, F and G reveal that a mutation within the putative ATP-binding motif of BALF3, which is responsible for nuclease activity [58], significantly reduced the numbers of micronuclei and phosphorylation of H2AX detected following wild-type BALF3 expression. On this basis, the genetic alterations of cells caused by EBV BALF3 may be attributed to its nuclease activity.

Phosphorylated H2AX is a mediator in response to DNA damage, which recruits DNA repair proteins at the chromatin lesion to carry out repair, and it has been reported to be associated with numerous cancers, including oral squamous cell, esophageal, breast, non-small cell lung, gastric, bladder, colorectal carcinoma, as well as NPC [71-79], suggesting that DNA damage occurs and may play a role in the development of cancers although this correlation remains to be understood. Previously, EBV infection was found to induce H2AX phosphorylation in NPC cells through its reactivation [55]. In this study, NPC cells exhibited γH2AX by expression of EBV lytic gene product BALF3 and aggravated genomic instability upon recurrent BALF3 expression. In addition, cancer is considered a consequence of accumulation of molecular damage via multiple replication and selection events [80], suggesting that the progressive tumorigenic features in NPC cells may be due to accumulation of non-random genomic instability induced by BALF3. Moreover, although BALF3 triggered the DNA damage response (DDR), phosphorylation of H2AX, no conspicuous cytotoxicity and inhibition of cell proliferation were shown in host cells in the long-term experiments. Regarding DDR, γH2AX is not only a biomarker for DNA damage but also the induction of senescence and mTOR overactivation, which is defined as pseudo-DDR [81]. The atypical DDR is induced by various stimuli, such as senescence and DNA replication stress, which can be suppressed by blockage of mTOR activity [81-83]. Furthermore, combination of activation of the mTOR pathway and avoidance of cell cycle arrest account for carcinogenesis [80]. In NPC cell lines and tissues, factors involved in the mTOR pathway, such as PI3K and AKT, are commonly activated [84-87] and mTOR inhibitors are validated and evaluated for clinical therapies [88-91]. Accordingly, it is also a possibility that activation of the mTOR pathway might take place in NPC cells after BALF3 expression, in addition to low-dose DNA damage, which lead to tumor progression.

Fluctuations of anti-EBV VCA IgA antibody titers can be seen in healthy individuals and these increase before the onset of NPC, as well as in patients with relapse of NPC [62, 92, 93], revealing that EBV reactivation occurs periodically in nasopharyngeal epithelial tissues. To imitate the reactivation of EBV in nature, in our previous study, NPC cell lines bearing the EBV genome were treated with 12-*O*-tetradecanoylphorbol-13-acetate (TPA) and sodium butyrate (SB), known inducers of the EBV lytic cycle, through repeated cycles of lytic induction and recovery, resulting in remarkable genomic instability and tumorigenic phenotypes of NPC cells [55]. Thus, in this study, the recurrent expression protocol (Figure 3A) for examining EBV BALF3 in NPC cells was designed in accordance with the natural process and our previous study. Moreover, in the long-term experiments, the expression of BALF3 in TW01TREx-BALF3 cells was induced by DOX at a concentration of 5 ng/ml. At this concentration of DOX, the level of BALF3 mRNA was up to approximately 30.2% of the maximal BALF3 level in the EBV-positive NPC cell line, NA cells, treated with 40 ng/ml TPA and 3 mM SB for 36 h, as determined by a quantitative reverse transcription-PCR analysis (data not shown). This indicates that a lower level of BALF3 expression was examined in this study and this may represent early or incomplete viral reactivation. This led to dramatic effects on the cells but did not generate apparent cytotoxicity detectable by observations of cell growth and ordinary cell culture under these conditions. On the other hand, comparing the effects of recurrent EBV BALF3 expression and EBV reactivation induced repeatedly by 40 ng/ml TPA and 3 mM SB on NPC cells [55], the effect of whole virus expression gives a greater degree of genomic instability and tumorigenic phenotypes than expression of BALF3 alone, suggesting that the induced expression of EBV BALF3 in this study at lower levels, as discussed above, or EBV BALF3 might cooperate with other viral gene products in the EBV-mediated promotion of NPC relapse.

The malignant features of NPC progressed with recurrent expression of EBV BALF3 and the various differentially expressed cancer-related genes identified above may be involved in this progressive development. The gene expression profile acquired by RNA expression array analysis includes 172 altered genes associated with the features of cancer and 62 of these genes were consistent with the results of array CGH, excluding their expression levels relative to the control (Supplementary Table S1 and Figure 6A and B) and implying that some indirect contributory factors might be involved in the regulation of gene expression, such as mutation within a regulatory region, RNA and protein stability and chromosome aberrations. We also identified 5 overexpressed oncogenes in both arrays, *EVI1*, *FIGF*, *PAK1*, *SOX2* and *TP63* (Figure 6C). EVI1, ecotropic viral integration site 1 [94], is a transcription factor that regulates hematopoietic differentiation, cell proliferation and cell death and is highly expressed in myeloid leukemia and epithelial cancers. FIGF, c-fos induced growth factor, or called vascular endothelial growth factor D [95, 96] is a secreted glycoprotein that activates VEGF receptor (VEGFR)-2 and -3 on the surface of epithelial cells to drive the formation of blood vessels and lymphatics. PAK1, p21-activated kinase 1 [97], is a downstream effector of GTP-bound Rho GTPase Cdc42 and Rac, activation of which stimulates cytoskeletal reorganization, cell survival, cell proliferation, cell migration and anti-apoptosis. SOX2, sex determining region Y-box 2 [98], is a transcription factor that modulates the pluripotency and self-renewal of embryonic stem cells and the early development of tissues, as well as the cell proliferation, cell invasion and apoptosis of several solid tumors. TP63, tumor protein p63 [99], functions as a transcription factor to regulate p53 target genes and is associated with the cell cycle, DNA damage repair and apoptosis in epithelial cancers. Among these oncogenes, *EVI1*, *FIGF*, *SOX2* and *TP63* have been reported in and are correlated with NPC [100-103], suggesting that EBV BALF3 enhances their expression in the process of NPC relapse. Thus, *PAK1* is also believed to be a potential biomarker for diagnosis of NPC although this remains to be confirmed.

Although EBV infection has been known for many years to be closely associated with NPC, how EBV contributes to NPC carcinogenesis remains to be elucidated. It has been suggested that latent EBV may participate in the carcinogenesis of NPC via high-grade preinvasive lesions of nasopharyngeal cells [104]. Moreover, we have shown that the EBV latent gene product LMP1 and EBNA2 induce genomic instability in epithelial cells [45, 48]. Considering that almost 100% of people living in NPC high-risk regions carry latent EBV, but only a few individuals develop NPC, latent infection seems not to be the only scenario. Retrospective, cross-sectional and prospective studies indicate that individuals with high levels of antibodies against EBV lytic antigens, markers of EBV reactivation, tend to have a higher risk of developing NPC [19, 21, 23, 25, 92, 93, 105, 106], suggesting that the EBV lytic cycle also may play an important role in the carcinogenesis of NPC. Furthermore, the presence of LOH can be detected in histologically normal nasopharyngeal epithelia and the cases increase dramatically in the population of NPC patients whether in high or low-risk regions [107, 108]. In addition, the titers of IgA antibodies against EBV lytic antigens increase prior to the development of NPC [19-21, 25, 28, 93, 109], implying that genomic instability emerges at an earlier time and contributes to the initiation of NPC, in which EBV reactivation is involved. Thus, we compared the effects of latency and the lytic cycle of EBV on the genomic instability of NPC cells and found that recurrent reactivation of EBV gives rise to a profound enhancement of the tumorigenic properties of the cells, in addition to promoting genomic instability [55]. Among the EBV lytic gene products, we have shown that EBV DNase and BGLF4 are able to induce genomic instability in NPC cells [56, 57]. We provide evidence here that BALF3 is another EBV lytic gene product contributing effectively to the induction of genomic instability and the consequent tumorigenicity of NPC cells. Therefore, the findings of this study might be applied to the elucidation of NPC carcinogenesis. Because blocking BALF3 retards the replication of EBV [58], it is possible that EBV BALF3 is as another target for preventing the development of NPC.

## MATERIALS AND METHODS

### Cell lines

NPC-TW01 (TW01) and HONE-1 are human nasopharyngeal carcinoma cell lines, derived from the tumor of a Taiwanese patient and a Chinese patient, respectively, which have lost the EBV genome [110, 111]. TW01 and HONE-1 cells were cultured in Dulbecco's modified Eagle's medium (Thermo Fisher Scientific) supplemented with 10% fetal bovine serum (Biological Industries) and incubated at 37°C and 5% CO_2_. TW01TREx-VC and TW01TREx-BALF3 cells were established from TW01TREx cells, TW01 cells express stably the tetracycline repressor, transfected with pLenti4 (Invitrogen) and pLenti4-BALF3, respectively, and selected by 25 μg/ml blasticidin (Sigma-Aldrich) and 500 μg/ml zeocin (Invitrogen). TW01TREx-VC and TW01TREx-BALF3 cells are TW01TREx cells harboring pLenti4 and pLenti4-BALF3, respectively, and were cultured in Dulbecco's modified Eagle's medium supplemented with 10% tetracycline-free fetal bovine serum (Invitrogen) and incubated at 37°C and 5% CO_2_. In addition, the selected clones were maintained by supplementation with 12.5 μg/ml blasticidin and 250 μg/ml zeocin. For the induction of BALF3 expression, TW01TREx-BALF3 cells were treated with doxycycline (DOX) (Sigma-Aldrich).

### Transfection

Cells were transfected with plasmids or small interfering RNAs (siRNAs) using transfection agents according to the manufacturer's instructions (Invitrogen). Briefly, for the expression of EBV BALF3 in TW01 cells, the cells were transfected with the indicated concentration of pEGFP-C1 (Clontech Laboratories) or pEGFP-C1-BALF3 using Lipofectamine 2000 (Invitrogen) at 37°C for 4 h and the culture medium was refreshed prior to incubation at 37°C for 24 h. For the establishment of Tet-regulated BALF3 inducible cell lines, TW01TREx cells were transfected with 3 μg pLenti4 or pLenti4-BALF3 using Lipofectamine 2000 at 37°C for 4 h and the culture medium was refreshed, followed by overnight incubation at 37°C and antibiotic selection. For the knockdown of EBV BALF3, TW01TREx-BALF3 cells were transfected with the indicated concentration of Stealth RNAi siRNA specific to BALF3 or Negative Control Duplexes (Invitrogen) by siPORT *NeoFX* (Invitrogen) at 37°C for 24 h and then treated with DOX for 24 h after replacement of fresh culture medium. The siBALF3s used in this study were siBALF3-1 (5'-CACUGGCAUCUAGCCAGCAAAUUCU-3') and siBALF3-2 (5'-GCCGCCUUUGAGAAUUCCAAGU ACA-3').

### Western blotting

Cell extracts were harvested by radioimmunoprecipitation (RIPA) buffer (50 mM Tris [pH7.5], 150 mM NaCl, 0.1% SDS, 10 mM EDTA, 1% Igepal CA-630 and protease inhibitor cocktail [Roche Applied Science]) for 20 min on ice and then centrifuged at 15,000 × *g* for 10 min at 4°C to collect the supernatants. The lysates were mixed with bromophenol blue buffer and then heated at 95°C for 5 min. The samples were separated by sodium dodecyl sulfate-polyacrylamide gel electrophoresis (SDS-PAGE) at 100 V and then transferred onto a nitrocellulose membrane (GE Healthcare) at 300 mA for 90 min in a cold room. The membrane was soaked in 5% skim milk at room temperature for 1 h. After blocking, the membrane was incubated with primary antibodies specific to green fluorescent protein (GFP) (Clontech Laboratories), V5 (Invitrogen), γH2AX (Cell Signaling) and glyceraldehyde-3-phosphate dehydrogenase (GAPDH) (Biodesign) at 4°C overnight prior to horseradish peroxidase-conjugated goat anti-mouse IgG (Jackson ImmunoResearch Laboratory) at room temperature for 1 h. The signal was detected by development with an enhanced chemiluminescence substrate (PerkinElmer) and exposure to X-ray film (Fujifilm).

### Micronucleus assay

Micronucleus assay was carried out as described previously [55] with some modifications. Slide-cultured cells were fixed in 4% paraformaldehyde at room temperature for 20 min and then permeabilized in 0.1% Triton X-100 at room temperature for 5 min. DNA was stained with Hoechst33258 (Sigma-Aldrich) at room temperature for 30 s and the slides were covered with a mounting medium (Vector Laboratories). For the evaluation of micronucleus occurrence, more than 1 × 10^3^ fixed cells were examined and photographed using a fluorescence microscope (Olympus) and the data were expressed as percentages.

### Indirect immunofluorescence staining

Slide-cultured cells were fixed in 4% paraformaldehyde at room temperature for 20 min and then permeabilized in 0.1% Triton X-100 at room temperature for 5 min. The fixed and permeabilized cells were blocked in 4% fetal bovine serum at room temperature for 30 min and incubated with the anti-γH2AX antibody (Upstate) at room temperature for 1 h prior to the incubation of Rhodamine Red-X-labeled goat anti-mouse IgG (Jackson ImmunoResearch Laboratory) at room temperature for another 1 h. DNA was stained with Hoechst33258 at room temperature for 30 s. The slides were covered with mounting medium and photographed using a fluorescence microscope.

### Cell proliferation assay

Cells were seeded onto 96-well plates at 1 × 10^3^ cells/well and incubated at 37°C and 5% CO_2_ for 0, 24, 48, 72 and 96 h. After incubation, WST-1 reagent (Roche) was added to the culture medium and incubated for 2 h. The dye solution produced by metabolically active cells was quantified by an ELISA reader (Tecan) with absorbance at 440 and 650 nm.

### Recurrent expression of EBV BALF3

TW01TREx-VC and -BALF3 cells were seeded and treated with 5 ng/ml DOX at 37°C and 5% CO_2_ for 24 h and then the culture medium was refreshed prior to incubation for another 24 h, which is defined as one passage (P). After incubation, the cells were trypsinized and reseeded for the next passage. In this study, the periodic induction was performed up to 15 passages and the cells were used for further studies.

### Array-based comparative genomic hybridization (array CGH)

Array CGH was carried out according to the manufacturer's instructions (Agilent Technologies). Briefly, genomic DNA was purified using a DNeasy Tissue Kit (Qiagen) and subjected to SurePrint G3 Human CGH Microarray Kit 1 × 1M and TW01TREx-BALF3 without DOX treatment at passage 1 was used as the common reference in this study. Following hybridization and washing steps, the array slides were scanned and features were obtained by Feature Extraction (Agilent Technologies). The extracted features were subsequently analyzed by Agilent Genomic Workbench version 7.0.4.0 (Agilent Technologies). The enrichment of aberrant regions was determined using the Z-score detection algorithm with a moving average window of 5 Mb and the threshold of algorithm was set to a value of 2.5 to generate aberration calls for each altered locus [54].

### Cell migration assay

Cell migration assay was carried out according to the manufacturer's instructions (Platypus Technologies). Briefly, cells were seeded onto 96-well plates containing Oris stoppers and incubated at 37°C and 5% CO_2_ overnight. After incubation, the stoppers were removed and the cells were incubated for 12 h to permit cell migration. The images were photographed by a optical microscope (Olympus). The area of a cell-free zone was measured by ImageJ (National Institutes of Health) and cell migration was presented as percent closure, which was calculated using the following formula: [(pre-migration)_area_ — (migration)_area_/(pre-migration)_area_] × 100.

### Cell invasion assay

Cell invasion assay was carried out as described previously [55] with some modifications. Briefly, the membranes of HTS FluoroBlok inserts (BD Biosciences) were coated with Matrigel Basement Membrane Matrix (BD Biosciences) and cells were resuspended in 2% tetracycline-free fetal bovine serum-containing DMEM and loaded into the upper chambers, which were placed in 24-well plates with 10% tetracycline-free fetal bovine serum-containing DMEM, and then incubated at 37°C and 5% CO_2_ for 12 h. After incubation, the cells on the membrane were fixed with methanol at 4°C for 15 min and stained with 5 μg/ml propidium iodide (Sigma-Aldrich) at room temperature for 10 min. The signal was detected and photographed using a fluorescence microscope to count the number of invaded cells.

### Spheroid assay

Cells were seeded onto 0.7% or 1% agarose-coating 96 well plates and incubated at 37°C and 5% CO_2_ for 21 days. After incubation, the images were photographed using an optical microscope.

### In *vivo* tumorigenesis assay

All animal experiments in this study were carried out according to the Guide for the Care and Use of Laboratory Animals of the National Institutes of Health. Seven-week-old non-obese diabetic/severe combined immunodeficiency (NOD/SCID) female mice were used in this study and 2 × 10^6^ cells were resuspended in serum-free DMEM and injected subcutaneously into the right dorsal flank. The mice were monitored weekly and tumor size was measured using callipers. The tumor volume was estimated using the following formula: volume = length × width^2^ × 0.52. Sacrifices were performed at week 6 and the tumors were removed and weighed.

### RNA expression analysis

RNA expression analysis was carried out according to the manufacturer's instructions (Affymetrix). Briefly, RNA was purified by RNeasy Mini Kit (Qiagen) and single-stranded cDNA was generated using a WT cDNA Synthesis Kit (Affymetrix) and then fragmented and labeled with WT Terminal Labeling Kit (Affymetrix). The samples were hybridized with GeneChip Human Gene 2.0 ST Array and scanned with a GeneChip scanner 3000 7G (Affymetrix). Raw data were processed with Partek Genomics Suite 6.6 (Partek) for normalization and gene level analysis. Gene ontology was determined using the Database for Annotation, Visualization and Integrated Discovery (DAVID; http://david.abcc.ncifcrf.gov) [112, 113].

## SUPPLEMENTARY FIGURES AND TABLE


