# Supplementary material for: Epstein-Barr virus BALF3 mediates genomic instability and progressive malignancy in nasopharyngeal carcinoma
Source: Oncotarget. 2014 Aug 10;5(18):8583–601. doi: 10.18632/oncotarget.2323 (PMC4226706; doi:10.18632/oncotarget.2323)
Supplement: Supplementary file 1 [file oncotarget-05-8583-s001.pdf]

SUPPLEMENTARY FIGURES AND TABLE

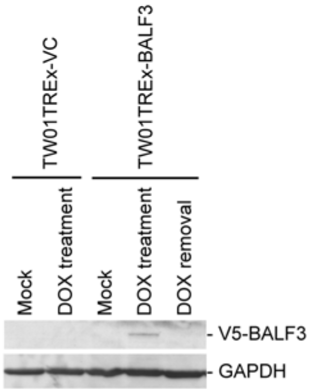

Supplementary Figure S1: Examination of EBV BALF3 expression in NPC cells. TW01TREx-VC and TW01TREx-BALF3 cells were treated with 5 ng/ml DOX for 24 h and the culture medium of TW01TREx-BALF3 cells at 24 h post-treatment was refreshed and incubated for another 24 h. The protein extracts of all groups were subjected to western blotting with antibodies specific to V5 and GAPDH. Mock, mock treatment; DOX, doxycycline.

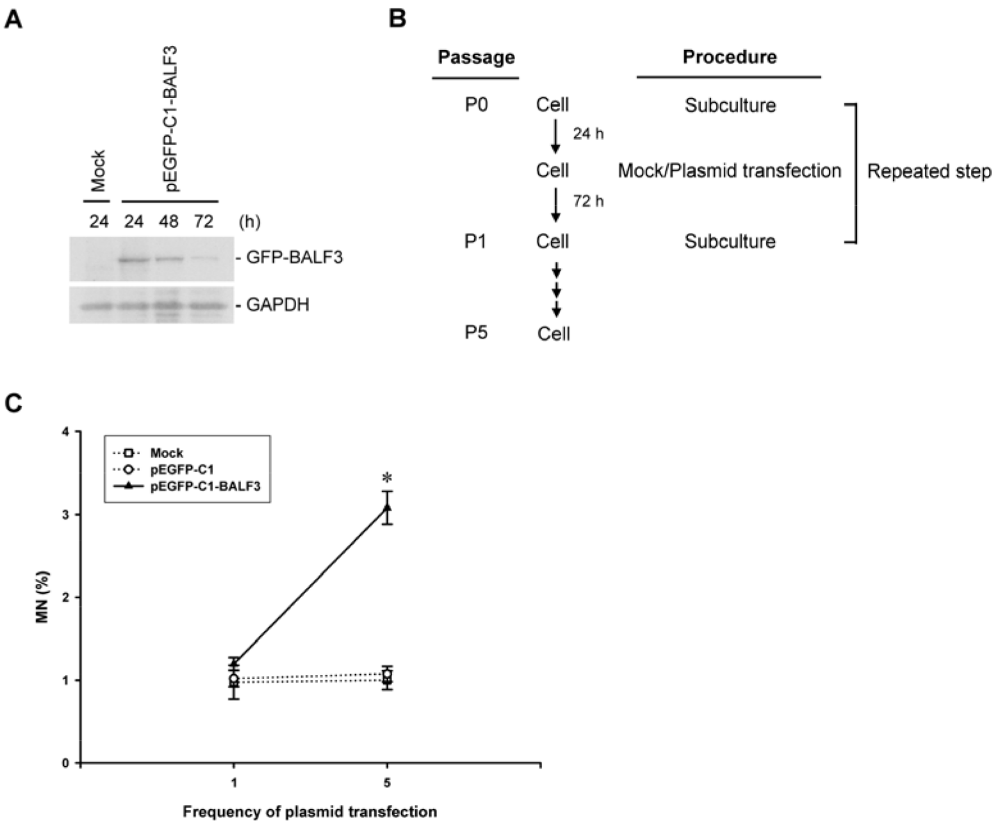

Supplementary Figure S2: Accumulation of genomic instability in NPC cells after recurrent EBV BALF3 expression. **(A)** HONE-1 cells were transiently transfected with 0.2 µg pEGFP-C1-BALF3 for 24, 48 and 72 h. The protein extracts of all groups were subjected to western blotting with antibodies specific to GFP and GAPDH. Mock, mock transfection. **(B)** Representative illustration of recurrent EBV BALF3 expression in NPC cells. The recurrent EBV BALF3 expression protocol was as follows. Cells were seeded on a culture plate and incubated for 24 h, followed by mock transfection or transfection with 0.2 µg pEGFP-C1 or pEGFP-C1-BALF3 for 72 h. After incubation, the cell subculture was performed for the next round. These above procedures were defined as one passage (P) and repeated. The cycle was carried out up to 5 rounds. **(C)** The cells were harvested at passages 1 and 5 and subjected to micronucleus assay. Data are presented as means ± standard deviations. Student's *t* test was used to determine the difference between two groups. *, $P < 0.01$, compared to HONE-1 cells with pEGFP-C1 transfection. Mock, mock transfection; MN, micronucleus.

Supplementary Table S1. Gene ontology of genes differentially expressed in NPC cells after recurrent BALF3 expression[a]

GO term[b]	Gene count	*P* value	Gene symbol[c]
Regulation of cell adhesion	40	4.7E-2	*CLEC7A*, ***EGFL6***, ***FAT4***, ***KAL1***, *PDZD2*, ***PPFIBP1***, *SLAMF7*, *TEK*, ***AMTN***, ***CDH24***, *CCL5*, *CLDN4*, ***COL3A1***, *COL4A6*, ***DSC3***, ***DSG3***, *ENG*, ***FLRT3***, *FNDC3A*, *GNE*, ***HES1***, *HAPLN1*, *IGFBP7*, ***ITGA6***, *ITGBL1*, *ICAM1*, *LY6D*, *MUC16*, *MUC4*, ***PKP1***, ***PDPN***, *PTPRM*, ***PCDH7***, ***PCDHB14***, *PCDHB2*, ***SIRPA***, *SPOCK1*, ***SUSD5***, *TNXA*, *TNXB*
Regulation of cell death	37	1.2E-1	*ADAMTSL4*, *ARHGEF9*, *FGD1*, ***KCNIP3***, *PYCARD*, *RASGRF2*, *TRAF5*, *XAF1*, ***ALDOC***, ***ANG***, ***ATXN10***, *BIRC3*, *BIRC7*, *CARD6*, *DAPK1*, ***DNASE1L3***, ***EYA2***, *FGF2*, ***GJA1***, *INPP5D*, *IFI6*, *IL24*, *IL6*, ***IFT57***, *KLK8*, *KRT18*, ***LGALS1***, ***MAPK8***, *NIPA1*, ***PAK1***, *PMAIP1*, *PSEN2*, *PML*, ***RNF144B***, *SULF1*, *TGFB2*, ***TP63***
Regulation of immune system	31	5.8E-4	*CLEC7A*, *DDX58*, *CCL5*, ***COL3A1***, *C1R*, *C4BPB*, *C4A*, *C4B*, ***CFHR5***, *CFI*, *DPP4*, *INPP5D*, *ICAM1*, *IL10*, *IL13RA1*, *IL2RG*, *IL29*, *IL6*, *IL6ST*, *IL7*, *IL7R*, *PLCG2*, *PSEN2*, *PML*, ***PRKCQ***, *SCIN*, *C3*, *TGFB2*, *TNFSF13B*, *ZFP36*, ***ZNF675***
Regulation of cell migration	26	1.2E-1	***EPHA4***, ***KAL1***, ***KLF7***, *APBB2*, ***ANG***, *ASZ1*, *CATSPER4*, *CCL5*, *DNAH11*, *ENG*, *FGF2*, ***ITGA6***, *ICAM1*, *IL10*, *IL6*, ***MAPK8***, ***NTF3***, *PLAU*, *PDGFRB*, *PODXL*, ***PTGS2***, *PTPRM*, *SPOCK1*, *TGFB2*, *TGFBR3*, *VEGFC*
Regulation of cell growth	20	1.1E-1	***ADRB2***, *APBB2*, *CTH*, *CDA*, *DAB2*, *FLVCR1*, *FGF2*, ***GAMT***, *CACNA1C*, *IGFBP6*, *IGFBP7*, *IL7*, *MCTS1*, *NOV*, ***NRG1***, ***PPARG***, *PML*, ***PRKCQ***, *TGFB2*, ***TP63***
Regulation of vasculature development	19	1.9E-1	*CITED2*, *KLF4*, ***SOX2***, ***ANG***, *DYRK1B*, *ENG*, ***EPGN***, ***EYA2***, *FGF2*, *LEPR*, ***MMP2***, *PLAU*, *PSEN2*, *PTPRM*, *TGFB2*, *TGFBR3*, *TRIM15*, ***TP63***, *VEGFC*
Generation of precursor metabolites and energy	16	3.0E-1	*ATP5EP2*, *ATP6V1B1*, *EDARADD*, *NDUFA1*, *ALDH5A1*, ***ALDOC***, ***FRRS1***, *GFPT2*, ***GYG1***, *HKDC1*, *IL6ST*, *LEPR*, *ME3*, ***PHKA2***, *PDHB*, *SLC1A3*
Regulation of defense response	16	7.8E-4	*NT5E*, *CLEC7A*, *DDX58*, ***ADRB2***, *ACE2*, *CCL5*, *IL10*, *IL6*, *IL6ST*, ***PPARG***, ***PLA2G4A***, *PML*, ***PTGS2***, *C3*, *UACA*, *ZFP36*
Regulation of cell cycle	16	3.7E-1	***BRIP1***, ***CDC45***, *CITED2*, *FOSL1*, ***RAD9A***, *APBB2*, *BTC*, ***CDKN2B***, ***EPGN***, ***FAM5B***, *HERC5*, ***HOXA13***, *PML*, ***PTGS2***, ***PRKCQ***, *TGFB2*
Regulation of DNA repair	16	1.9E-1	***BARD1***, ***BRIP1***, ***BTG2***, ***DCLRE1B***, ***FANCB***, ***FANCE***, ***RAD9A***, ***CHAF1B***, *CUL4B*, ***EYA2***, *POLN*, ***POLA1***, *PML*, ***RFC2***, ***RFC4***, ***RNF168***
Response to DNA damage	6	2.3E-1	***BRIP1***, ***RAD9A***, *NUPR1*, *PML*, ***TP63***, *UACA*

Abbreviations: GO, gene ontology.
[a]TW01TREx-BALF3+DOX (P15) was compared to TW01TREx-BALF3 (P1). The gene expression profile was analyzed by GeneChip Human Gene 2.0 ST Array and the gene ontology was determined using DAVID.
[b]GO terms in this list are related to capabilities necessary for cancer.
[c]The overexpressed genes are presented in boldface.
